# Relating Trait Variation to Species and Community Productivity in Contrasting Oro-Mediterranean Pastures: A 7-Years Study in the Pollino National Park (S-Italy)

**DOI:** 10.3390/plants11192471

**Published:** 2022-09-21

**Authors:** Domenico Gargano, Giuseppe Fenu, Nicodemo G. Passalacqua, Aldo Schettino, Liliana Bernardo

**Affiliations:** 1 Department of Biology, Ecology and Earth Sciences, Università della Calabria, Via P. Bucci, I-87036 Rende, Italy; 2 Dipartimento di Scienze della Vita e dell’Ambiente, Università degli Studi di Cagliari, Viale Sant’Ignazio da Laconi, I-09123 Cagliari, Italy; 3 Ente Parco Nazionale del Pollino, Complesso Monumentale Santa Maria della Consolazione, I-85048 Rotonda, Italy

**Keywords:** biodiversity conservation, climate change, functional traits, Mediterranean mountain vegetation, plant productivity

## Abstract

Understanding how functional traits influence community assemblage and functioning is crucial for assessing the effects of global change on vegetation composition. We studied the functional composition (i.e., plant size (SIZE), leaf area (LA), specific leaf area (SLA), and leaf dry matter content (LDMC)) of a xerophile pasture and a mesophile grassland in southern Italy, and recorded species richness (SR), plant cover (COV) and flowering rates (FLOW) over a 7-year period. Both communities revealed the dominance of stress-tolerators, probably reflecting an adaptation to the Mediterranean climate. The functional classification of species distinguished three groups. Species from the mesophile community had larger SIZE and LA, while those from the xerophile pasture showed higher LDMC; SLA was not connected to the source community. Community-level analyses confirmed such patterns, but with higher SLA in the mesophile grassland. While SR was comparable, COV and FLOW varied between the communities. At the species level, LDMC was positively related to FLOW and the inter-annual variability of COV and FLOW. At the community level, SIZE, LA and SLA were positively related to COV, while LDMC was positively related to FLOW. Trait variations can significantly contribute to the xerophile–mesophile shift in Mediterranean mountain vegetation, by regulating the productivity of species and communities in the two contexts and, possibly, their responsiveness to global change.

## 1. Introduction

The Mediterranean Basin is one of the most important global biodiversity hotspots [1,2]. Despite a relatively narrow surface, this region harbors an extraordinary floristic richness, mainly due to its marked ecological heterogeneity [2,3]. Especially, the occurrence of high mountain systems, and related ecological gradients, makes a major contribution to the plant diversity of the Mediterranean area [2,3]. A large fraction of the oro-Mediterranean floristic richness occurs in pasture and grassland communities, which qualify as relevant biodiversity hotspots [4]. To date, these communities have been included among the most threatened ecosystems because of the impact of ongoing global change on the biodiversity of south European mountain areas [5]. The novel regimes of temperature and rainfall promoted by climate change are expected to modify previous patterns of plant distribution, according to the physiological tolerance of species to the newly established climate conditions [6]. Accordingly, significant floristic rearrangements have occurred on European mountain systems, where thermophile species are expanding their latitudinal and altitudinal ranges by replacing the microtherm ones [7]. This process is accompanied by a relevant loss of species richness in south European mountain ranges [8].

Further components of global change, such as land-use change, are also inducing significant biodiversity alterations in herbaceous Mediterranean mountain communities. In fact, in Mediterranean mountain systems, most of the ongoing land-use/land-cover changes are determined by current grazing patterns, which are promoting different trajectories of vegetation dynamics depending on local ecological and management conditions [9]. However, the overall decline of extensive grazing practices in marginal mountain areas is promoting rapid processes of reforestation. Such floristic and vegetation variations have important implications for the current, as well as for future, socio-ecological and conservation scenarios. Indeed, the global change-driven effects on the biodiversity and functioning of pasture and grassland ecosystems can affect crucial ecological processes that depend on the interplay of vegetation composition and local climate conditions, such as litter decomposition rates [10] and other soil fertility traits [11], among other things. This would result in a general reduction in the ecosystem services provided by these mountain ecological systems [12,13]. Furthermore, the species range variations caused by global change are likely to reduce the future effectiveness of current networks of protected areas for preserving crucial species and ecosystems [14].

Therefore, understanding how species-rich communities cope with environmental heterogeneity is needed for building up reliable expectations regarding the influence of global change on future biodiversity patterns, a crucial requirement for improving global conservation strategies. Plant responses to environmental changes depend on a complex set of abiotic and biotic parameters, including soil resource availability, which in turn depend on regional and local topo-edaphic heterogeneity [15,16,17] and community features (e.g., species richness) [12]. By virtue of the spatial and temporal variability affecting the modulators of plant response to ecological variations, the global change effects on species and communities can vary substantially across ecological contexts and vegetation types (e.g., [17,18]).

Mediterranean mountain pastures and grasslands show complex vegetation patterns depending on a wide array of ecological and human drivers. Among these, the transition from xeric to mesic conditions is responsible for relevant floristic and vegetation shifts, and its effects can be observed at regional [19] as well as local scales [20]. Concerning the mesophile/xerophile shift, it is generally thought that the elevated trophic availability of fertile soils can increase inter-specific competition, resulting in a reduced species richness compared to communities established in xerophile contexts [21]. Instead, the xerophile pastures, generally having greater species richness, appear less stable at the species population level, and this can favor a higher likelihood of local extinctions [22,23]. On the other hand, fertile grasslands can experience more severe drought constraints [24]. A higher sensitivity of fertile grasslands to resource restrictions can result from the abundance of species possessing a high relative growth rate [25], and this can be further exacerbated by the management regime they are subjected to [26].

Traditionally, studies on the responsiveness of plant communities to environmental changes rely on species richness as a main modulator of community resistance and resilience against ecological variations [27,28]. Accordingly, species richness has been found to modulate grassland responses to the variation in relevant ecological drivers, including climate [26,29], resource availability [29,30] and management intensity [26,29]. The main justification for the positive relationship between biodiversity and ecosystem stability lies in the fact that a greater amount of species may provide a larger extent of between-species compensation by mitigating the effects of ongoing perturbations (i.e., portfolio effect). Nonetheless, some studies have shown no or negative relationships between biodiversity and stability components in grassland ecosystems. For instance, contrarily to the above-mentioned general expectation, Pfisterer and Schmid [31] found that resistance and resilience to perturbations can be higher in species-poor communities than in rich ones. A similar outcome was found by van Ruijven and Berendse [32], in whose study species diversity appeared to be related to the resilience, but not to the resistance, of grasslands against drought stress. Some deviation from the general biodiversity/stability relationship can be explained by the fact that species within a community are not functionally equivalent. The occurrence (or the depletion) of a particular species or functional group may affect the stability of the whole community independently of its overall richness [31]. Moreover, the loss of species playing a key role in facilitative interactions involving co-adapted taxa could diminish the community response to a stress factor to a larger extent than expected [33]. This would also explain outcomes such as those found in Isbell and Wilsey [34], who showed that only the biodiversity component constituted by native species can contribute to stabilizing the functioning of grasslands under different grazing pressures. Such considerations highlight the need to consider the functional plant sphere for improving our understanding of the patterns linking community composition and dynamics to environmental variability [18,35,36,37]). Especially, the traits showing clearer links to plant productivity appear particularly informative, because primary productivity proxies (e.g., above-ground biomass, plant cover) are generally retained as robust estimators of ecosystem functioning. Accordingly, Diaz et al. [38] demonstrated that most of the global plant trait variation is expressed in a two-dimensional space, where one dimension is related to the size of the whole plant, while the other represents the variation in leaf economics traits. Together, size and leaf economics traits can express how the plants cope with the growth limitations occurring in their habitats [39,40,41].

The marked intra- and inter-annual climatic variability, and the striking contrast between dry and mesic sites, make Mediterranean mountain areas an optimal place for investigating the responses of plant species and communities to environmental heterogeneity. In this paper, we evaluated the relationships between the functional composition and the productivity rates expressed over a 7-year timeframe in two contrasting (xerophile vs. mesophile) herbaceous communities established in the Southern Apennine (S-Italy). The studied communities represent habitats of strategical interest for conservation in the European Community, and, despite high and fairly comparable species richness, reveal striking floristic differences due to inter-site variations in local climate, topography, soil features, and seasonal dynamics [42]. This has offered the opportunity for evaluating the possible relationships between community functional and productivity traits under different ecological conditions. To this end, the functional measures and productivity data recorded from 2012 to 2018 were analyzed to evidence patterns of functional differentiation among species and communities, and to relate the observed functional patterns to the productivity rates expressed at the species and community levels over the 7-year study period. More specifically, we aimed to: (1) provide a coherent trait-based species classification and assess the contribution of each trait to the species functional differentiation; (2) evaluate the community-level effects of patterns of species functional differentiation; and (3) investigate the possible relationships between functional features and patterns of plant cover and flowering.

## 2. Results

### 2.1. Species Functional Classification and Trait Contribution to Species Functional Differentiation (Q1)

The functional traits were not significantly correlated, except for leaf area (LA, hereafter) with plant size (SIZE hereafter; r = 0.487; *p* < 0.001). The CSR classification highlighted a predominance of stress-tolerators, as 74 species (89.2% of the total) revealed a prevalent S strategy, while the species with prevalent R or C strategies numbered 5 and 4, respectively (see Table S1 for a complete report on CSR classification). The correlation analyses between CSR classification and functional measures shows that the percent of C strategy was positively related to SIZE (ρ = 0.248, *p* = 0.02) and LA (ρ = 0.944, *p* < 0.001). Instead, the S score was negatively related to LA (ρ = −0.477, *p* < 0.001) and specific leaf area (SLA, hereafter; ρ = −0.814, *p* < 0.001), and positively related to leaf dry matter content (LDMC, hereafter; ρ = 0.305, *p* = 0.005). Finally, the percent of R strategy was positively related to SLA (ρ = 0.923, *p* = 0.001) and negatively related to LDMC (ρ = −0.357, *p* = 0.001).

**Figure 1:**
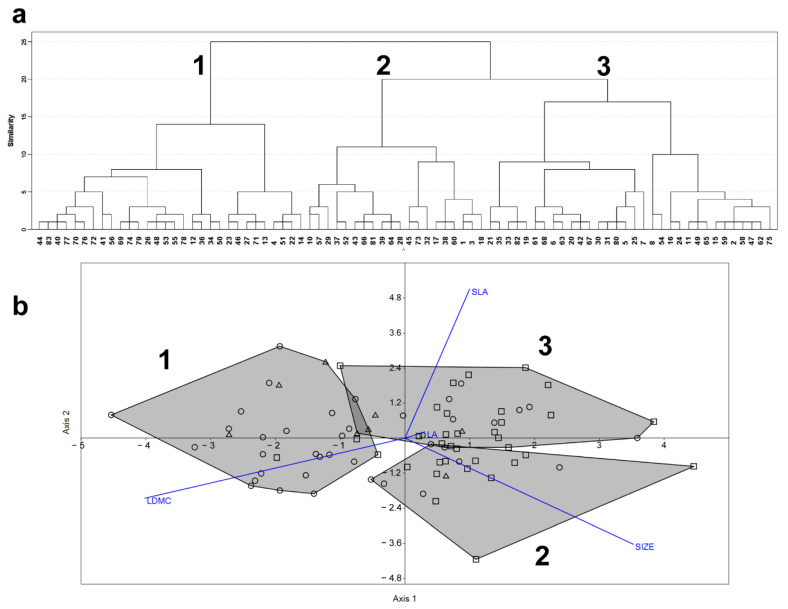
Functional groups obtained by the numerical classification of 82 plant species based on four functional traits (**a**) and discriminant analysis performed to test the reliability of the functional groups (**b**). Biplot symbols: circles, species exclusive to the xerophile community; squares, species exclusive to the mesophile community; triangles, species found in both communities.

The classification of the species functional matrix produced three main functional groups (FGs, hereafter; Figure 1a) whose compositions are shown in Table S1. The discriminant analysis confirmed that 92.7% of the attributions emerged from the cluster analysis; the classification reliability slightly varied across FGs, ranging from 84.4% to 100.0% (Table 1). According to the between-trait correlation patterns, the four functional traits gave rise to three independent gradients that were related to the FGs (Figure 1b). All the functional traits varied significantly among FGs (SIZE: F = 27.8, *p* < 0.001; SLA = 12.3; *p* < 0.001; LDMC: F = 28.3, *p* < 0.001), with the only exception being LA (F = 0.103; *p* = 0.902).

**Figure 2:**
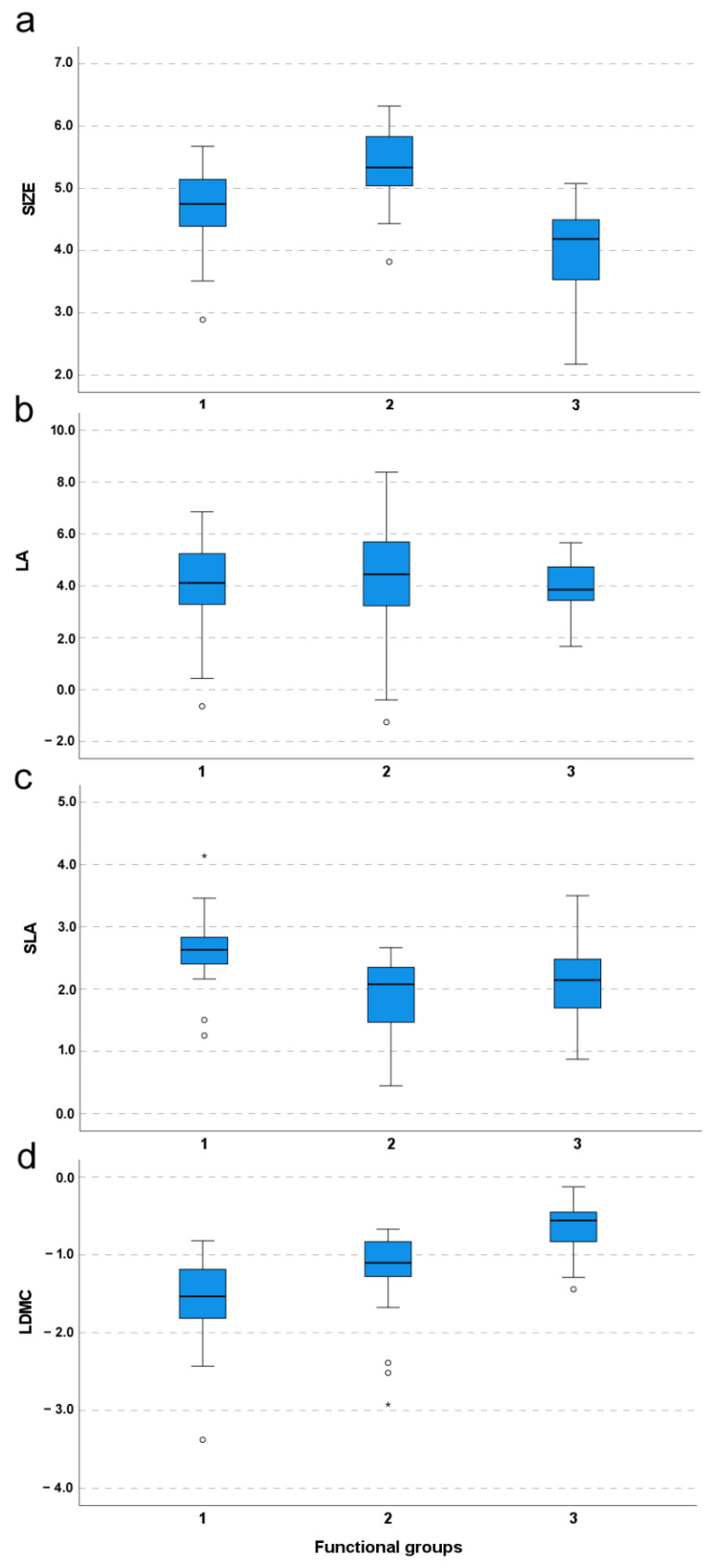
Variation patterns of the four functional traits ((**a**) = SIZE; (**b**) = LA; (**c**) = SLA; (**d**) = LDMC) across the functional groups (FGs) produced by the cluster analysis as presented in Figure 1a. All values have been log-transformed. Abbreviations: SIZE = plant size; LA = leaf area; SLA = specific leaf area; LDMC = leaf dry matter content.

As shown in Figure 2c, the FG3 was characterized by higher SLA compared to both FG2 (difference = 0.80; *p* < 0.001) and FG1 (difference = 0.51; *p* = 0.003), while no significant differences occurred between FG2 and FG3 (difference = −0.29; *p* = 0.26). The plant species belonging to the FG2 were distinguished by a larger size compared to both FG3 (difference = 0.65; *p* = 0.002) and FG1 (difference = 1.36; *p* < 0.001); in addition, the plants assigned to FG1 were significantly smaller than ones included in FG3 (difference = −0.71; *p* < 0.001) (Figure 2a). As shown in Figure 2d, the species falling in FG1 possessed higher LDMC compared to FG3 (difference = 0.94; *p* < 0.001) and FG2 (difference = 0.61; *p* < 0.001); however, no significant differences in LDMC occurred between FG3 and FG2 (difference = −0.32; *p* = 0.07). Finally, pairwise comparisons did not reveal significant between-group differences of LA (FG3-FG2: *p* = 1.00; FG1-FG3: *p* = 1.00; FG2-FG1: *p* = 1.00) (Figure 2b).

### 2.2. Community-Level Effect of Species Functional Differentiation (Q2)

**Figure 3:**
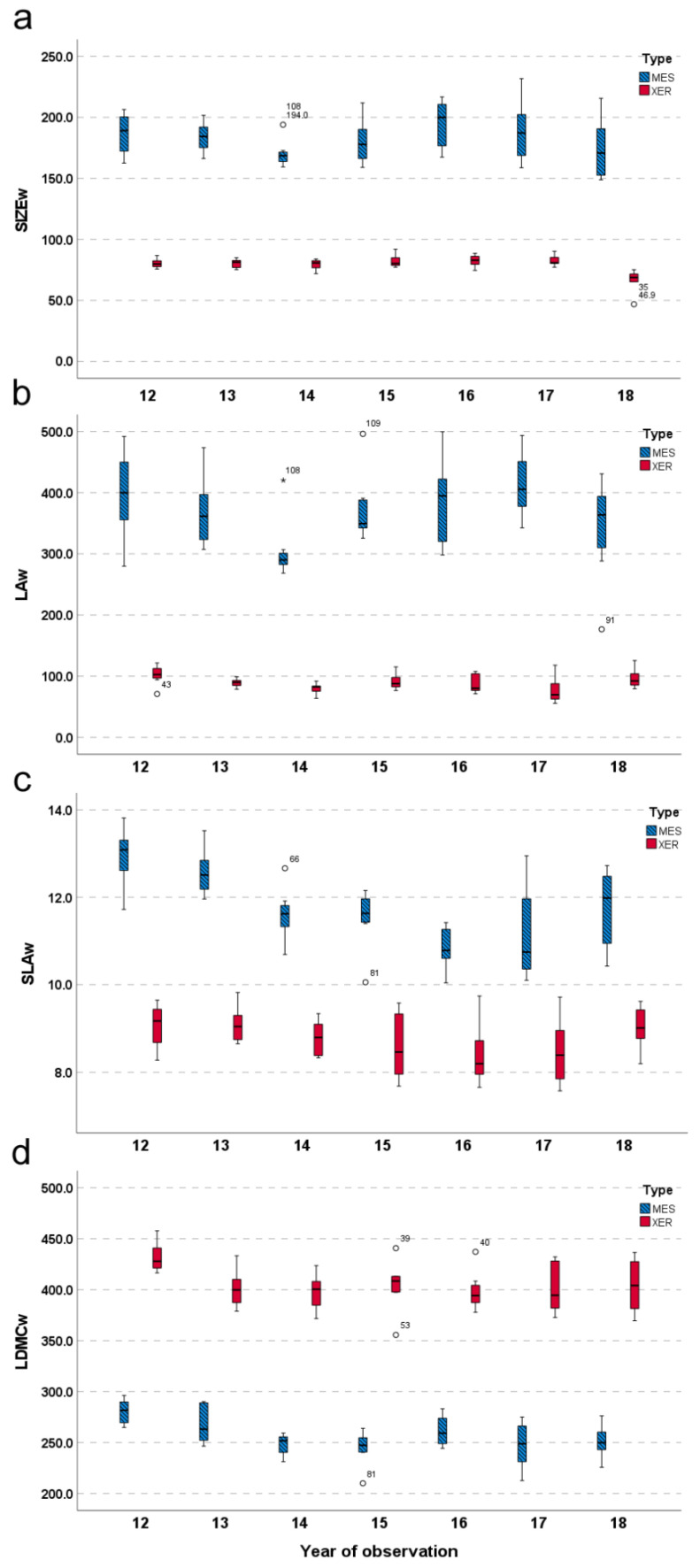
Variation patterns of community-weighted traits over the 7-year study period in the mesophile (MES) and xerophile (XER) communities. Abbreviations: SIZEw = community-weighted plant size (**a**); LAw = community-weighted leaf area (**b**); SLAw = community-weighted specific leaf area (**c**); LDMCw = community-weighted leaf dry matter content (**d**).

The patterns of species functional classification were congruent with the relative source community (Pearson χ^2^ = 26.138; *p* < 0.001). In detail, the FG3 included 45.5% and 39.4% of species from the mesophile and xerophile communities, respectively, along with a minor fraction (15.1%) of species occurring in both communities (Figure 1). The FG2 was mainly constituted by species exclusive of the mesophile community (81.0%), while the FG1 was dominated by species found in the xerophile community only (79.3%). At the species level, the mesophile community revealed larger average values of plant size (t = 37.646; *p* < 0.001; Figure 3a) and leaf area (t = 30.251; *p* < 0.001; Figure 3b) whereas the xerophile community showed higher average values of LDMC (t = −37.455; *p* < 0.001; Figure 3d). Conversely, SLA played a minor role in differentiating species with regard to the community of origin. At the community level, this trait reached higher values in the mesophile community compared to the xerophile one (t = 19.595; *p* < 0.001; Figure 3c).

### 2.3. Investigating Relationships between Functional and Productivity Patterns of Species and Related Communities (Q3)

The regression model implemented on the whole species sample evidenced an overall negative relationship between plant size and flowering rates (Table 2), while the LDMC was positively related to patterns of inter-annual variability of species cover, and flowering and to the average species flowering rate (Table 2). Such results suggest that LDMC was the major modulator of species productivity in the studied system, with a major emphasis on inter-annual variations in species cover and flowering. However, the regression models involving xerophile and mesophile species separately indicate that some relationships between species’ functional and productivity traits could be habitat-dependent. Indeed, in the mesophile species group, the average species cover was positively affected by LDMC, and especially by LA (Table 2). Instead, by considering the species within the xerophile community, we confirmed a positive relationship between LDMC and the inter-annual variability of species cover (Table 2). 

**Figure 4:**
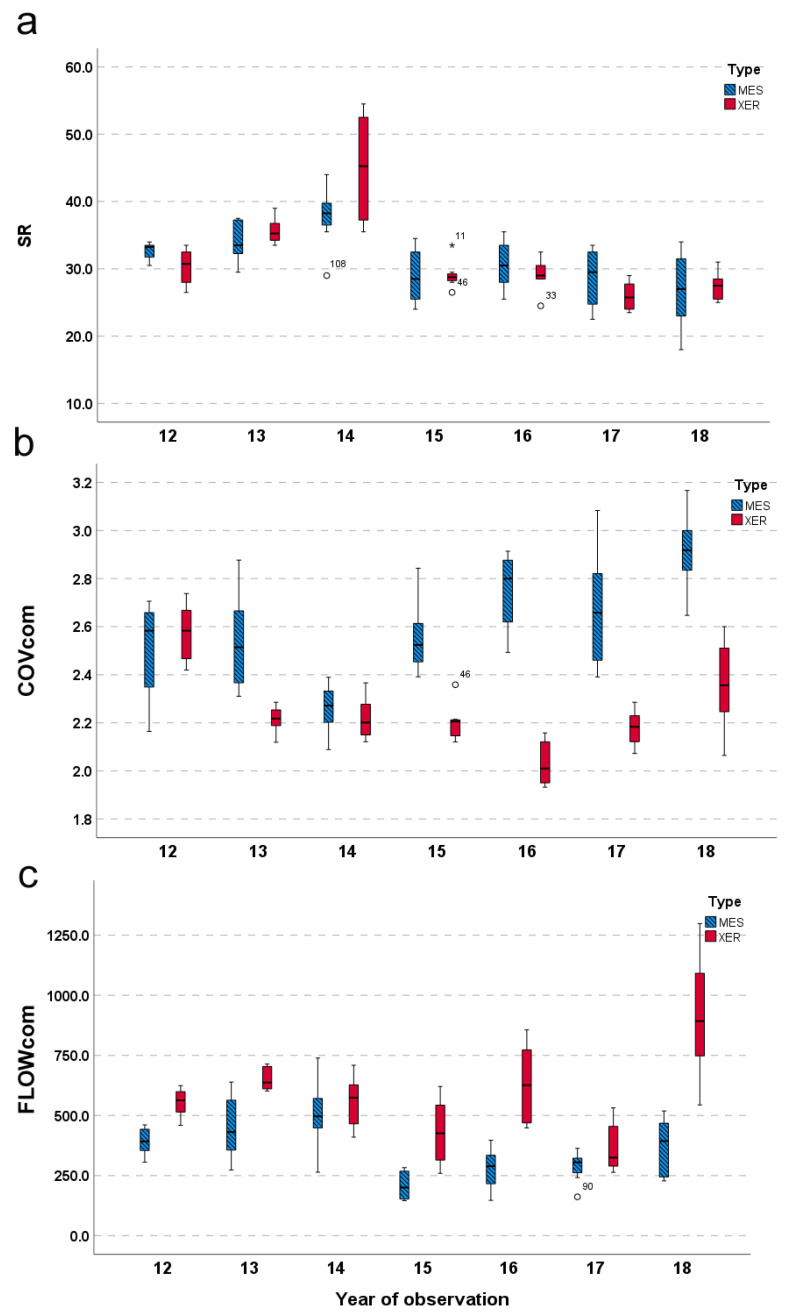
Variation patterns of community productivity proxies over the 7-year study period in the mesophile (MES) and xerophile (XER) communities. Abbreviations: SR = no. of species found in each plot (Species richness) (**a**); COVcom = the sum of all species cover estimated for each plot (plant cover) (**b**); FLOWcom = sum of all flowered individuals/ramet counted in each plot (Flowering rate) (**c**).

At the community level, the average SR over the 7-year interval ranged between 35.13× sampling unit in the mesophile site and 36.50× sampling unit in the xerophile one, with no significant differences between the two communities (t = 0.14; *p* = 0.89; Figure 4a). Instead, the average COV recorded in the mesophile community (2.60) was higher than in the xerophile one (2.25), and this difference was significant (Mann–Whitney test: D = 0.607; *p* = 0.001; Figure 4b). On the contrary, the average annual flowering rate was significantly higher in the xerophile pasture than in the mesophile grassland (640.05 ± 203.24 and 440.15 ± 127.50, respectively; t = 2.96; *p* < 0.05; Figure 4c).

**Figure 5:**
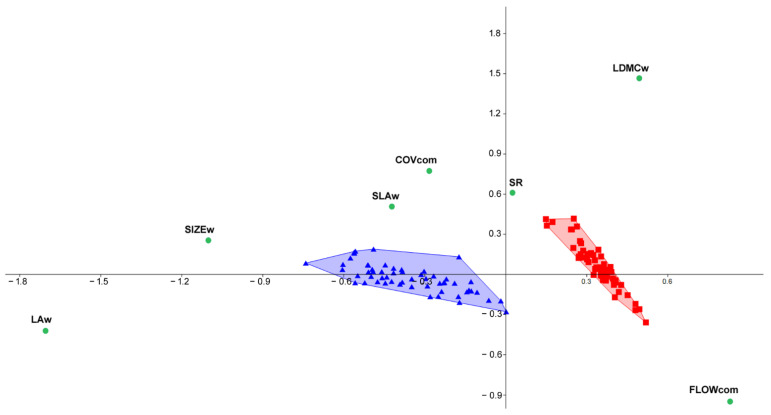
Correspondence analysis carried out on the 112 vegetation samples based on community functional and productivity traits. Symbols: triangles, mesophile plot; squares, xerophile plots; dots, community-weighted functional traits and community productivity proxies.

The community-level analyses involving functional and productivity data confirmed the same patterns observed in the species data. The correspondence analysis clearly distinguished the vegetation samples from different communities. In the mesophile community, greater cover was associated with increasing scores of SIZEw, LAw, and SLAw (Figure 5). On the contrary, in the xerophile community, higher flowering rates (FLOWcom) were positively related to LDMC (Figure 5).

Overall, the community-weighted functional traits did not show any connection with species richness; nonetheless, by separating the data relative to the two communities, increasing values of plant size and leaf area appeared linked to a significant decrease in species richness in the mesophile plots (Table 3). The analyses carried out on the overall dataset indicate that the community-weighted values of plant size, leaf area, and specific leaf area were positively related to community cover (Table 3), while the same traits showed a negative correlation with the community flowering rates (Table 3). On the contrary, the community-weighted leaf dry matter content was positively and negatively linked to community flowering and cover, respectively (Table 3). 

Again, the partial datasets highlighted that such patterns incurred some between-community differences. For instance, while in the mesophile samples the community-level plant size showed a positive relationship with vegetation cover, in the xerophile one, the opposite trend was observed (Table 3). A similar contrasting pattern was found in the relationships between community-level LDMC and plant cover. In this case, LDMC was not linked to cover in the mesophile samples, while the same functional trait showed a positive correlation with the vegetation cover in the xerophile dataset (Table 3).

## 3. Discussion

### 3.1. Functional Characterization of Species and Communities (Q1–2)

The adopted classification and validation framework [43] indicates that the considered traits significantly contributed to categorizing the 83 plant species into three main functional groups (FGs), which are well sustained from a statistical viewpoint. All the traits significantly varied across FGs, confirming the relevance of plant size and leaf economics features to the functional distinction of plants [38]. As far as the leaf traits are concerned, the direction of the relative gradients (Figure 1b) suggested a little inter-dependence between SLA and LDMC, which is consistent with the different functions of these two traits [44,45]. Overall, the species were displaced over a SIZE-SLA vs. LDMC gradient (Figure 1b), which agreed with the theoretical expectations. The size and leaf economics traits reflect a trade-off between a rapid growth rate (i.e., high SIZE and SLA, small LDMC) and a conservative use of primary resources (i.e., small SIZE and SLA, high LDMC) [38,44]. Therefore, the frequency of functional types showing small SIZE and SLA and high LDMC should increase under more severe growth restrictions. Indeed, in resource-poor habitats, the long-term persistence of plants would require the improvement of their potential for stress tolerance rather than competitive ability [39,40].

The xerophile/mesophile shift is a major driver of regional and local floristic differentiation in oro-Mediterranean herbaceous communities, and it represents a typical transition from nutrient-poor to nutrient-rich habitats [19,20]. The studied communities are a clear example of the large floristic distinctiveness that can occur between Mediterranean xerophile pastures and mesophile grasslands [42]. Taxonomically, such a distinctiveness was well expressed in the sample of species considered for functional analyses, as only 8 out of 83 taxa (<10%) occurred in both the communities. However, because variations in climate and soil fertility exert relevant effects on plant traits at global and local scales (e.g., [37,41]), the community-level effects of the xerophile/mesophile ecological transition should also reflect the relationships between species traits and growth conditions. In our data, the species’ ordination over the functional space and their ecological preferences fitted such an expectation, suggesting that the considered functional traits provide services with respect to specific environmental conditions [46]. Accordingly, most of the “mesophile” and “xerophile” taxa were associated, respectively, to large SIZE and high LDMC (Figure 2). Nonetheless, the level of between-community functional distinction was lower than the taxonomic one. Most of the mesophile/xerophile variation was explained over a gradient of large SIZE–low LDMC to small SIZE–high LDMC (Figure 1b(2)), whereas SLA produced an intermediate gradient related to FGs, including a mixture of species from the two contrasting communities (Figure 1b(2)). This intermediate behavior of SLA-related taxa would have contributed to the taxonomical and functional distinction between the two communities. Although SLA is traditionally considered a promoter of plant growth [44,45], in our study system, it was substantially independent of plant size, and appeared related to a more flexible ecological behavior. 

Further reasons for the functional overlap observed between the two communities could be related to the regional ecological and management context. Indeed, the summer drought typical of Mediterranean climate regions can favor the inclusion of taxa with thermo-xerophile traits also in sites showing mesophile soil properties [42]. This could be interpreted as a process of biogeographically driven thermophilization, showing some analogy to the thermophilization induced by climate warming [7]. In addition, the intense grazing pressure occurring in the mesophile site may have further contributed to increasing the observed functional similarity between the two communities. Indeed, plants adapted to disturbed environments typically possess a greater tolerance to drought and tissue destruction, conditions that may select for a short life-time and high reproductive output [40,47,48]. For this reason, under intense management regimes, plant communities are expected to shift along the fast–slow continuum, defined by their growth strategies [49]. Moreover, our data suggest that a certain degree of functional convergence may have also contributed to improving the species diversity in the mesophile site. Indeed, fertile grasslands are generally expected to be more productive but poorer in species than xeric pastures [21]. Nonetheless, the studied communities revealed a comparable extent of species richness. Hence, the Mediterranean mountain grasslands can derive a biodiversity benefit from their complex functional composition.

### 3.2. Linking Functional Features to Inter-Annual Productivity Patterns (Q3)

Plant size and leaf economics traits are closely related to the productivity challenges that plants experience in the wild [18,37,39,40]. In particular, local environmental variations are expected to enhance filtering processes that promote the segregation of species with different trait combinations into different communities [41]. Accordingly, the observed relationships between functional and productivity proxies indicate that the measured traits were relevant modulators of plant productivity in the two analyzed communities. Overall, our data evidence a general trade-off between functional traits and growth and flowering functions, as we found a clear association between size traits (i.e., SIZE, LA, and SLA) and plant cover, while LDMC was mainly related to flowering rates. Such association patterns had an evident link with the community type. Higher cover appeared to be related to the functional traits proper of species and functional groups predominant in the mesophile community, fitting the overall rule that higher soil fertility promotes the acquisition of competitive traits and results in an increase in primary productivity [25,39]. Instead, higher flowering rates and higher inter-annual variability in both cover and flowering rates were related to the functional traits proper of species predominant in the xerophile community. Other authors have already highlighted that xerophile pastures are more unstable due to their higher turnover affecting species and populations in such species-rich assemblages [22,23]. Nonetheless, our findings offer a way to evaluate, as the functional composition, rather than the mere species richness, can be related to the stability of community productivity patterns. Because LDMC is widely recognized to indicate a conservative use of primary resources [38,40,44], the positive relationship with inter-annual variations in plant productivity observed in our study highlights the adaptive role of this trait, which can improve plants’ abilities to cope with limited and unpredictable patterns of resource availability. Overall, the species richness was barely related to plant traits. However, increasing size traits and plant cover appeared to be linked to a relevant loss of plant diversity in the mesophile community. This finding emphasizes the sensitivity of grassland communities against variations in resource and management regimes [26,29].

Traditionally, species diversity qualifies as a major modulator of community responses to environmental perturbations (e.g., [12,27,28,30]). Nonetheless, various studies have evidenced relevant deviations from the classical biodiversity/stability relationship [31,32]. For instance, the loss [33] or acquisition [34] of species with high or low ecological value for the reference community can induce effects that diverge from those expected under a general higher biodiversity/higher stability assumption. Likewise, given the comparable plant diversities recognized in the two communities included in our study [42], the different productivity patterns observed over the 7 years cannot be justified by differences in species richness, but depend on a different combination of plant traits and productivity regimes. The xerophile community appeared more resilient to variations in resource availability because the host species are functionally equipped to cope with enhanced variability in cover and flowering rates. Contrarily, the species-rich mesophile grasslands relied on stable and high productivity rates. This would make such community types more sensitive to the declining trophic availability caused by increasing aridity and the exasperated management regime [24,26]. Therefore, the biodiversity consequences of ongoing rainfall reduction [50,51] and high grazing pressure [9] for Mediterranean mountain grasslands need to be carefully monitored.

In conclusion, our results allow us to characterize the xeric/mesic shift from a functional perspective, and to evaluate whether the observed interplay between functional and productivity patterns can suggest different responsiveness to global change components in species and communities with diverse functional and ecological requirements.

## 4. Materials and Methods

### 4.1. Study Sites

Fieldwork was carried out in two study sites, hosting, respectively, a xerophile pasture and a mesophile meadow. The study sites lie in the Pollino National Park, a large (>1900 Km^2^) protected area in S-Italy. The two community types were chosen because the transition from xeric to mesophile habitats is a major driver of vegetation shift in Mediterranean mountains [19]. Moreover, they are listed as habitats of priority interest for conservation in the Annex I of the “Habitat” Directive (92/43/EEC) with the following codes: “6210–*Semi-natural dry grasslands and scrubland facies on calcareous substrates (Festuco-Brometalia)* (*important orchid sites)”, and “6510–*Lowland hay meadows (Alopecurus pratensis, Sanguisorba officinalis”*’. The xerophile pasture was on Mt. Serra (N 39.84804°; E 16.09311°; elevation 1300 m a.s.l.). The calcareous bedrock and the steep slope promoted a discontinuous plant cover due to the high frequency of outcropping rock, a typical structure of the rocky pastures established on the Apennine slopes [52]. The most common species include *Armeria canescens* Boiss., *Bromopsis erecta* (Huds) Fourr., *Festuca circummediterranea* Patzke, and *Poa alpina* L. However, such perennials are accompanied by numerous ephemeral taxa (e.g., *Bromus hordeaceous* L., *Parentucelia latifolia* (L) Caruel, *Medicago lupulina* L., and *Dasypyrum villosum* (L) Candargy). The mesophile community was found at Piano Ruggio (N 39.91197°; E 16.13053°; elevation 1600 m a.s.l.), within a large doline. Here, despite the calcareous bedrock, the local topography induces striking pedological and vegetation differences compared to Mt. Serra [42]. The soil is deep and has a prevalent loamy texture, which reduces water drainage and limits summer drought. The scarcity of outcropping rock allows for a continuous plant layer constituted by a variety of mesophile species typical of the grasslands established on the flat or depressed surfaces occurring in the Apennine mountain belt [53]. In this site, the most common species are *Achillea millefolium* L., *Agrostis capillaris* L., *Cynosurus cristatus* L., *Dactylis glomerata* L. subsp. *hispanica* (Roth.) Nyman, and *Festuca microphylla* (St.-Yves ex Coste) Patzke. It is noteworthy that this site hosts some rare endemic taxa (e.g., *Plantago media* L. subsp. *brutia* (Ten.) Arcang.), along with species close to their range border (i.e., *Gentiana lutea* L.). A previous study [42] highlighted that the communities possess a comparable species richness (~30 taxa/m^2^), but with a low amount of shared species.

### 4.2. Trait and Plant Sampling for Functional Measures

The functional measures were carried out on plants growing in the same area studied for obtaining productivity data. The selected traits were plant size (SIZE) and leaf area (LA), representing the general sizes of the plants, and specific leaf area (SLA) and leaf dry matter content (LDMC), related to leaf structure. Such traits are highly responsive to the ecological characteristics of growth sites [37,38,41]. The functional traits were measured on 10 individuals/ramet *per* species randomly selected in the field. For rare species occurring with less than 10 individuals/ramet, the functional measures included all the recognized individuals. Overall, we measured 712 individuals/ramet representing 83 plant species (i.e., 36 species from Piano Ruggio, 39 from Mt. Serra, and 8 species found in both communities). Because the estimated species richness for the two communities is close to 30 taxa/m^2^ [42], the taxonomic sample involved in functional analyses was congruous in order to represent the overall floristic spectrum of both communities. All traits were measured according to Cornelissen et al. [45]. Individual values of SLA and LDMC were obtained by averaging measures relative to two leaves per individual/ramet. Overall, whole individual samples were collected in the field, stored in nylon bags, and then measured in the laboratory within 24 h. However, for some large species such as *Gentiana lutea* and *Cirsium tenoreanum*, plant size (in mm) was measured in the field, while two leaves per plant were taken for laboratory measures as indicated above. In the laboratory, we first measured plant size and leaf fresh weight (i.e., 2 leaves per plant). Afterwards, the leaves were scanned, and the relative images were processed with ImageJ [54] to calculate LA (in mm^2^). Finally, the leaves were dried in an oven, and their dry weight was determined with a digital or an analytic balance depending on sample size and mass. SLA was determined as the ratio of leaf area to dried weight, expressed in mm^2^ mg^−1^. The LDMC (%) was obtained as the proportion between dried and fresh leaf mass expressed in mg. The complete list of measured taxa and relative functional measures is provided in Table S1.

### 4.3. Floristic Composition, Plant Cover and Flowering Rates

In the autumn of 2011, in each study site, 8 hexagonal permanent plots of about 3.2 m^2^ were delimitated over an overall surface of ca. 300 m^2^ for recording floristic data (i.e., species occurrence, cover and flowering rates).

Then, during the subsequent seven growing seasons (2012–2018), the two communities were monitored by field surveys aiming to record species richness, cover, and flowering rates in each sampling unit. The cover of each detected species was estimated based on the phytosociological method, then: r = negligible cover; + = cover < 1%; 1 = 1% < cover < 20%; 2 = 20% < cover < 40%; 3 = 40% < cover < 60%; 4 = 60% < cover < 80%; 5 = 80% < cover < 100%. The values of plant cover were assigned by averaging the estimation provided simultaneously by a team of two researchers who performed all the floristic surveys. Firstly, the vegetation data set was used to calculate the species richness (SR) expressed by the community in each year. Instead, before using plant cover data in statistical analyses, the phytosociological values assigned in the field were transformed according to the van der Maarel scale [55], where: r = 1, + = 2, 1 = 3, 2 = 5, 3 = 7, 4 = 8, and 5 = 9. The transformed values of species cover (COV) were then considered as a proxy of species abundance [56]. Flowering rates (FLOW) were quantified by counting all flowered individuals/ramet of each species encountered in each vegetation plot. Only for 2014, the number of flowering individuals was unavailable at the species level, and so it was recorded for the whole sampling unit. To limit the effects of inter-seasonal variations on the measured species productivity traits [42,57], the field surveys were repeated two times per year (June and July). The annual value of plant cover was then obtained by averaging the two estimations. Instead, the annual flowering rates of species and communities were calculated by summing up the two counts. Analogously, the annual value of species richness was taken to represent the overall number of species detected in each plot during the two seasonal field surveys.

### 4.4. Data Analysis

#### 4.4.1. Species Functional Classification and Trait Contribution to Species Functional Differentiation (Q1)

The individual functional measures were averaged to obtain a species-level functional matrix accounting for 83 plant species and 4 traits. In a preliminary step, the functional measures were log-transformed to meet normality. Then, the distribution of log-transformed data was checked by the Shapiro–Wilk test and by evaluating the relative levels of asymmetry and kurtosis. The normality tests indicated that the measures carried out on *Asphodelus macrocarpus* caused a substantial deviation from normality; as a consequence, the functional data of such plant species were excluded from subsequent analyses. The possible correlations between functional traits were tested by the Pearson correlation test. The species-level functional matrix permitted us to classify species according to two different approaches. The first one consisted in classifying species according to Grime’s CSR theory [39,47]. Therefore, the values of LA, SLA, and LDMC were used to determine the CSR ecological strategy of each species using the CSR strategy calculator tool provided by Pierce et al. [40]. Since the CSR classification procedure allows for assigning the percentage of each tertiary adaptive strategy to a given species, the relationships between species functional measures and tertiary strategy values were checked by Spearman’s rho correlation test.

For the second classification task, the species–traits matrix was used to perform a functional species classification by following the conceptual framework of Fry et al. [43]. Initially, the species were grouped into homogenous functional groups (FGs) by a hierarchical cluster analysis performed on the four log-transformed functional variables. Subsequently, the statistical support of the FGs was tested by discriminant analysis. Before multivariate analyses, data were standardized to limit noise due to differences in variability range among functional variables. Hence, the original values were transformed into dimensionless numbers and normalized following the *omnibus procedure* of Legendre and Legendre [58]. The cluster analysis was carried out using the paired group (UPGMA) agglomeration method with Pearson correlation coefficient as the distance measure. Then, the variation patterns of the three functional traits among the identified functional groups were evaluated by one-way ANOVA, adopting the Bonferroni *post-hoc* test for between-group comparisons.

#### 4.4.2. Community-Level Effect of Species Functional Differentiation (Q2)

At the community level, in a preliminary step, each of the eight species found in both communities was assigned to the community where it appeared to be more frequent. Then, the correlation level between the functional groups produced by the species functional classification of the source community (i.e., xerophile, mesophile) was tested by carrying out a contingency analysis for computing the Pearson’s chi-squared test (χ^2^). Moreover, to evaluate the level of between-community functional differentiation, we quantified the relevance of each functional trait in each community by adopting an approach similar to Bruelheide et al. [41]. Therefore, we weighted the contribution of each functional trait to the community cover by applying the following equation:

FTw = Ʃ (Fti × COVi)/COV(1)

where FTw = community-level functional trait weight (i.e., SIZEw, LAw, SLAw, LDMCw); FTi = functional trait value of the ith taxon; COVi = cover value of the ith taxon in the plot; COV = overall plant cover in the plot.

The between-community variations of functional trait weights were tested by Student’s *t*-test. 

#### 4.4.3. Linking Functional Features to Productivity Patterns of Species and Communities (Q3)

Further analyses aimed to evaluate the relationships between the functional features of species and communities and their inter-seasonal productivity patterns. The annual values of plant cover (COV) and number of flowered ramets (FLOW) allowed for determining four productivity proxies accounting for the average and variability of species cover and flowering over the observation period (respectively, 7 and 6 years for cover and flowering data). Such productivity proxies consisted in the average values of cover (COVave) and flowering (FLOWave), and in the variance of cover (COVvar) and flowering (FLOWvar) expressed by each species over the study timeframe. The original values of the productivity proxies were log-transformed to meet normality; the distribution of transformed data was checked by performing the Shapiro–Wilk test and evaluating levels of asymmetry and kurtosis. The possible relationships among species’ functional traits and measures of plant productivity were tested by partial regression models, assuming each proxy of plant productivity as a dependent variable to be regressed against the four species functional traits (i.e., SIZE, LA, SLA, and LDMC). To check for linearity and equality of variances, the standardized residuals were plotted against the standardized predicted values, and the observed outliers were removed from analyses; at the end of this process, the number of species included in each model ranged from 79 to 80, depending on the considered dependent variable. Three distinct regression models were run: one considered the whole species sample, while the other two models involved a species from each of the two communities.

At the community level, at the end of the fieldwork, the community-level data matrix accounted for 8 repeats × 2 sites × 7 years (N = 112). This data set was relative to four community-weighted functional traits (i.e., SIZEw, LAw, SLAw, LDMCw) and three productivity proxies: species richness (SR, no. of species found in each plot), plant cover (COVcom, the sum of all species cover estimated for each plot), and flowering rate (FLOWcom, sum of all flowered individuals/ramet counted in each plot). To avoid any possible effect due to dependence between the values of community cover and species richness, the values of COVcom were standardized by dividing by SR. The original values were log-transformed to meet normality. Between-community variations of SR, COVcom and FLOWcom were tested by Student’s t-test. The normality of each variable was tested by the Shapiro–Wilk (W) test. When the data deviated from normality, between-community productivity differences were evaluated by applying the Mann–Whitney nonparametric test (U). Overall community-level relationships between functional and productivity data were evaluated by a correspondence analysis, while community-level correlations between functional traits and productivity proxies were tested by the Pearson correlation test.

The statistical analyses were carried out using the software packages SPSS^®^ 27 for Windows (SPSS, Chicago, IL, USA) and PAST 3 [59].

## Figures and Tables

**Table 1:** Confusion matrix showing the attributions produced by the discriminant analysis (columns) with respect to the functional groups (FGs) obtained by the cluster analysis (rows) performed on the four functional traits. CC(%) = percent of correct classifications.

FG	1	2	3	Total	CC(%)
1	27	3	2	32	84.4
2	1	19	0	20	95.0
3	0	0	30	30	100.0
Total	28	22	32	82	

**Table 2:** Coefficients produced by the regression models carried out on log-transformed data of each measure of plant productivity (dependent variable) against the species functional traits (independent variables); separate models were ran by considering the whole species target and by distinguishing the species from different community type. ** coefficient significant at the 0.01 level; * coefficient significant at the 0.05 level. Abbreviations: COVave = average values of cover; FLOWave = average values of flowering; COVvar = variance of cover; FLOWvar = variance of flowering.

**All Plant Species**												
		**Measures of Species Productivity**										
		**COVave**			**COVvar**			**FLOWave**			**FLOWvar**	
Functional trait	N	*t*	*p*	N	*t*	*p*	N	*t*	*p*	N	*t*	*p*
SIZE	79	0.985	0.328	79	1.031	0.306	79	−2.052	0.04 *	80	−1.080	0.284
LA	79	1.057	0.294	79	1.146	0.256	79	−1.847	0.07	80	−1.392	0.168
SLA	79	−0.098	0.922	79	1.386	0.170	79	1.806	0.08	80	0.752	0.454
LDMC	79	1.301	0.197	79	3.105	0.003 **	79	2.267	0.03 *	80	2.113	0.04 *
**Mesophile plant species**												
		Measures of species productivity										
		COVave			COVvar			FLOWave			FLOWvar	
Functional trait	N	*t*	*p*	N	*t*	*p*	N	*t*	*p*	N	*t*	*p*
SIZE	36	0.267	0.791	36	1.758	0.089	33	−0.732	0.470	34	−0.215	0.831
LA	36	2.949	0.006 **	36	−0.647	0.523	33	−0.12	0.991	34	−0.165	0.870
SLA	36	0.058	0.954	36	0.346	0.732	33	0.448	0.657	34	−0.340	0.737
LDMC	36	2.298	0.028 *	36	0.711	0.482	33	1.176	0.250	34	0.761	0.453
**Xerophile plant species**												
		Measures of species productivity										
		COVave			COVvar			FLOWave			FLOWvar	
Functional trait	N	*t*	*p*	N	*t*	*p*	N	*t*	*p*	N	*t*	*p*
SIZE	43	0.042	0.967	43	0.000	1.000	45	−0.979	0.333	46	−0.515	0.610
LA	43	−0.657	0.515	43	1.625	0.112	45	−1.695	0.098	46	−1.290	0.204
SLA	43	−0.285	0.777	43	1.528	0.135	45	1.581	0.122	46	1.058	0.296
LDMC	43	0.549	0.586	43	2.518	0.016*	45	1.719	0.93	46	1.577	0.123

**Table 3:** Pearson correlation tests between community-level measures of functional traits and productivity proxies. Correlation tests were carried out on overall vegetation samples, and by separating the plots from different community types. ** correlation significant at the 0.01 level; * correlation significant at the 0.05 level. Abbreviations: SR = no. of species found in each plot (species richness); COVcom = the sum of all species cover estimated for each plot (plant cover); FLOWcom = sum of all flowered individuals/ramet counted in each plot (flowering rate); SIZEw = community-weighted plant size; LAw = community-weighted leaf area; SLAw = community-weighted specific leaf area; LDMCw = community-weighted leaf dry matter content.

**Overall Vegetation Data**									
		**Measures of Community Productivity**							
		**SR**			**COVcom**			**FLOWcom**	
Functional trait	N	*t*	*p*		*t*	*p*		*t*	*p*
SIZEw	112	−0.109	0.254		0.642 **	<0.001		−0.598 **	<0.001
LAw	112	−0.109	0.252		0.634 **	<0.001		−0.551 **	<0.001
SLAw	112	0.053	0.576		0.510 **	<0.001		−0.405 **	<0.001
LDMCw	112	0.010	0.913		−0.560 **	<0.001		0.516 **	<0.001
**Samples from the mesophile community**									
		Measures of community productivity							
		SR			COVcom			FLOWcom	
Functional trait	N	*t*	*p*	N	*t*	*p*	N	*t*	*p*
SIZEw	56	−0.549 **	<0.001	56	0.403 **	0.002	56	−0.412 **	0.002
LAw	56	−0.403 **	0.002	56	0.255	0.058	56	−0.283 *	0.034
SLAw	56	0.255	0.058	56	−0.248	0.066	56	0.286 *	0.032
LDMCw	56	−0.003	0.982	56	0.049	0.719	56	0.000	1.000
**Samples from the xerophile community**									
		Measures of community productivity							
		SR			COVcom			FLOWcom	
Functional trait	N	*t*	*p*	N	*t*	*p*	N	*t*	*p*
SIZEw	56	0.041	0.397	56	−0.299 *	0.025 *	56	−0.366 *	0.011
LAw	56	−0.061	0.653	56	0.070	0.607	56	0.162	0.232
SLAw	56	0.109	0.424	56	0.272 *	0.042	56	0.158	0.245
LDMCw	56	−0.122	0.370	56	0.266 *	0.048	56	−0.090	0.509

## Data Availability

Functional and productivity data relative to the 112 vegetation samples are available from the authors upon request.

## Supplementary Materials

The following supporting information can be downloaded at: https://www.mdpi.com/article/10.3390/plants11192471/s1, Table S1: List of plant species, community of origin, and functional and productivity measures used for statistical analyses.
